# Predictors of rhythm outcomes after cardiac resynchronization therapy in atrial fibrillation patients: When should we use an atrial lead?

**DOI:** 10.1002/clc.23527

**Published:** 2020-12-09

**Authors:** Sotirios Nedios, Michael Doering, Angeliki Darma, Johannes Lucas, Borislav Dinov, Arash Arya, Nikolaos Dagres, Gerhard Hindricks, Andreas Bollmann, Sergio Richter, Kerstin Bode

**Affiliations:** ^1^ Department of Electrophysiology Heart Center at University of Leipzig Leipzig Germany

**Keywords:** age, atrial fibrillation, atrial lead, cardiac resynchronization, ICD, LA diameter

## Abstract

**Background:**

Cardiac resynchronization therapy (CRT) is widely used in atrial fibrillation (AF) patients and could impact rhythm stability.

**Hypothesis:**

We aimed to identify predictors of sinus rhythm (SR) stability or AF progression in a real‐word cohort of CRT‐AF patients.

**Methods:**

From 330 consecutive implantable cardioverter‐defibrillator implantations due to ischemic or dilated cardiomyopathy, 65 (20%) patients with AF history (paroxysmal, *n* = 32) underwent a CRT implantation with an atrial electrode and were regularly followed every 4–6 months. Rhythm restoration was attempted for most AF patients based on symptoms, biventricular pacing (BP), and lack of thrombi.

**Results:**

After 33 months, 18 (28%) patients progressed to permanent mode switch (MS≥99%) and 20 (31%) patients had stable SR (MS < 1%). Logistic regression showed that history of persistent AF (OR: 8.01, 95%CI: 2.0–31.7, *p* = .003) is associated with higher risk of permanent MS. In persistent AF patients, a bigger left atrium (OR: 1.2 per mm, 95%CI: 1.03–1.4, *p* = .025) and older age (OR: 1.15 per life‐year, 95%CI: 1.01–1.3, *p* = .032) were predictors of future permanent MS. Paroxysmal AF at implantation (OR: 5.96, 95%CI: 1.6–21.9, *p* = .007) and increased BP (OR: 1.4 per 1%, 95%CI: 1.05–1.89, *p* = .02) were associated with stable SR. In persistent AF patients, stable SR correlated with higher BP (98 ± 2 vs. 92 ± 8%, *p* < .001).

**Conclusion:**

In patients with AF undergoing CRT implantation, persistent AF, LA dilatation and advanced age relate to future permanent MS (AF), whereas high BP promotes SR stability. These findings could facilitate the management of CRT‐AF patients and guide therapy in order to maximize its effect on rhythm.

**Abbreviations**
AF: atrial fibrillation
ATP: antitachycardia pacing
AV: atrio‐ventricular
BP: biventricular pacing
CI: confidence interval
CRT(D): cardiac resynchronization therapy (with a defibrillator)
ICD: implantable cardioverter‐defibrillator
ICM: ischemic cardiomyopathy
LA(D): left atrial (diameter)
LV‐EDD: left ventricular end‐diastolic diameter
LV‐EF: left ventricular ejection fraction
MS: mode switch
NIDCM: non‐ischemic dilated cardiomyopathy
NPV: negative predictive value
OR: odds ratio
PPV: positive predictive value
SD: standard deviation
SR: sinus rhythm
VF: ventricular fibrillation
VT: ventricular tachycardia
VV: inter‐ventricular

## 1 INTRODUCTION

Atrial fibrillation (AF) and heart failure (HF) are common and linked comorbidities. AF in implantable cardioverter‐defibrillator (ICD) recipients is associated with increased risk for mortality, hospitalization, and ventricular arrhythmias.^1^, ^2^, ^3^ Cardiac resynchronization therapy (CRT) has been shown to alleviate these effects and since HF patients today survive longer, CRT is increasingly being used in AF patients.^4^

However, the benefits of CRT therapy in AF patients are only comparable to patients in sinus rhythm (SR), if AF patients preserve a high level of effective biventricular pacing (BP).^5^, ^6^, ^7^ Improvement of left atrial (LA) function with reduction of AF burden and even restoration of SR has been reported in a small subset of patients with good CRT response and small LA size.^8^, ^9^, ^10^, ^11^ Although atrial and ventricular remodeling has a profound impact on clinical outcomes, many patients though experience no reverse remodeling at all and remain prone to AF progression.^12^

However, the factors that contribute to SR stability or AF progression after CRT have not been adequately evaluated yet. Therefore, the purpose of this study was to identify predictors of SR stability or future permanent AF in a real‐word cohort of CRT‐AF patients.

## 2 METHODS

### 2.1 Patients

Consecutive patients (*n* = 337) undergoing an ICD implantation during 2009–2011 were included in our institutional registry. Patients with hypertrophic‐obstructive cardiomyopathy (*n* = 5) or channelopathy (*n* = 2) were excluded, so that the final study population comprised of 330 patients with ischemic (ICM, *n* = 204, 62%) or non‐ischemic dilated cardiomyopathy (NIDCM, *n* = 126, 38%). ICM was defined as a reduced left ventricular ejection fraction (LV‐EF) associated with a significant coronary vessel obstruction, a history of myocardial infarction, or a history of coronary intervention. NIDCM was defined as a reduced LV‐EF in the absence of ischemic, hypertrophic, or other clear etiology of cardiomyopathy. AF was defined according to current guidelines as paroxysmal (self‐terminating, <7 days) or persistent (lasting >7 days or after a cardioversion) and permanent (accepted). The majority of CRT‐D recipients (*n* = 85/153, 56%) had no history of AF and were excluded from this study. Patients with permanent AF (*n* = 3) were excluded due to the lack of an atrial electrode. All patients with AF history (*n* = 65, 20%) and CRT implantation with an atrial electrode were included in the present study. Eligibility criteria for CRT were according to the current guidelines.^13^ All data were collected in accordance with the Declaration of Helsinki and the institutional research committee approved the study.

### 2.2 Echocardiography

Transthoracic echocardiography data were acquired prior to CRT implantation using a commercially available system (Vivid‐9 General Electric Vingmed, Milwaukee, USA) according to the guidelines of the American Society of Echocardiography. Left ventricular end‐diastolic diameter (LV‐EDD) and LA diameter were measured in the left parasternal long axis view. LV‐EF was calculated according to the Simpson method.

### 2.3 CRT‐D implantation

After a coronary sinus venogram, a bipolar LV pacing lead was inserted transvenously through the subclavian route with the help of an 8F‐guiding catheter preferably in a midventricular postero‐lateral position. After implantation of an atrial and right ventricular electrode, testing for pacing threshold/phrenic nerve stimulation and finally removal of the sheaths, all electrodes were connected to a CRT device. Device‐derived features that could support CRT delivery were activated when available. Post‐procedural X‐rays excluded a pneumothorax and ensured appropriate position of the leads. An electrical cardioversion was attempted in 22 patients at the time of implantation and in 17 patients within the first month.

### 2.4 CRT‐D programming and follow‐up

Device interrogation was performed at 4 weeks post implantation and then regularly (every 4–6 months) or on demand in an outpatient clinic. Beta‐blockers were up titrated according to blood pressure. Patients reporting an improvement of the NYHA class were classified as CRT responders. Echocardiographic atrioventricular (AV)/VV optimization was performed at the day after implantation and at follow‐up, if no clinical response was reported.

ICDs were programmed according to current recommendations for optimal detection and therapy.^14^ The ventricular fibrillation (VF) zone was typically set to >200 beats/min with at least 1 ATP prior to shock while the ventricular tachycardia (VT) zone was typically >170 beats/min with at least 3 ATPs prior to shock. The monitor zone was set to >150 beats/min and atrial arrhythmia detection to >170 beats/min with discriminators enabled.^15^ Two experienced physicians performed rhythm adjudications. An ICD therapy delivered for VT/VF was defined as appropriate, and all other episodes were deemed as inappropriate. ICD programming remained unchanged in all patients until therapies were delivered or an ablation procedure was performed, at which point patient‐specific programming changes were implemented. BP was calculated as the average of all available interrogations. If the mode‐switch was ≥99% after the CRT implantation despite rhythm control attempts, it was deemed as permanent (AF).

### 2.5 Statistics

Continuous variables are expressed as mean with SD if normally distributed (Kolmogorov–Smirnov test) or as median with interquartile range. Categorical variables are reported as proportions. Continuous variables were compared using the Student's *t* test (for normal) or the Mann–Whitney test (for non‐normal distribution), while categorical variables were compared using the chi‐square test. To determine the effect of SR on rhythm stability we detected patients that had mode‐switch <1% or > 99% in all interrogations following the CRT implantation and performed a logistic regression analysis to identify predictors of rhythm outcome. Analysis of patients with initial MS < 99% (requiring an atrial lead), but permanent AF during follow‐up was not aim of this study. Variables with a *p*‐value ≤.1 in univariate analysis were included in the multivariate model for determination of the odds ratio (OR) and its 95% confidence interval (CI). A *p*‐value of ≤.05 was considered statistically significant. Discrimination analysis identified the predictor values for the highest sensitivity and specificity. Accordingly, positive (PPV) and negative predictive values (NPV) were calculated. Analyses were performed using SPSS v20.0 (SPSS Inc., Chicago, IL, USA).

## 3 RESULTS

### 3.1 Patient characteristics

From 65 patients with AF history (32 with paroxysmal AF) who received a CRT with an atrial electrode, 18 (28%) patients progressed to permanent AF with continuous mode‐switch (≥99%) and 20 (31%) patients had stable SR (MS < 1%) during 33 (18–40) months of follow‐up. Most patients were in SR at implantation (*n* = 43, 66%). Some of these patients (*n* = 17) had an AF recurrence and underwent a cardioversion with a high success rate (88%, 15/17).

The rest of the patients with AF at implantation (*n* = 22/65, 44%) were offered a cardioversion within a month. Some patients had a persistent LAA thrombus (*n* = 4) or did not wish a cardioversion (*n* = 1), but 15 of 17 (88%) had a successful conversion in SR with a median time to recurrence of 80 (12–350) days. Therefore, most patients underwent a systematic effort to restore SR with cardioversion and sustain it with antiarrhythmic drugs or left atrial ablation (Table 1). One patient underwent AV node ablation. One patient died at 13 months and one underwent heart transplantation at 22 months of follow‐up.

**TABLE 1:** Characteristics of CRT‐D patients with and without permanent AF during follow‐up

	Total	Permanent AF at follow‐up		*p*
Number of patients, *n*	65	Yes (18)	No (47)	
Age, (years)	69 ± 8	71 ± 8	68 ± 8	.15
Males, *n* (%)	51 (79)	16 (89)	35 (75)	.31
Body mass index, kg/m^2^	29 ± 8	28 ± 9	29 ± 8	.83
Diabetes mellitus, *n* (%)	22 (34)	7 (39)	15 (32)	.77
Hypertension, *n* (%)	54 (83)	16 (89)	38 (81)	.71
Persistent AF, *n* (%)	33 (50)	15 (83)	18 (38)	.002
NYHA Class II, *n* (%)	23 (35)	10 (56)	13 (28)	.11
NYHA Class III, *n* (%)	35 (54)	7 (39)	28 (59)	.23
NYHA Class IV, *n* (%)	7 (11)	1 (6)	6 (13)	.85
Cardioversion, *n* (%)	34 (52)	13 (72)	21 (45)	.06
Amiodaron/Sotalol, *n* (%)	29 (45)	8 (44)	21 (45)	1.00
LA ablation for AF, *n* (%)	6 (9)	—	6 (13)	.18
AV node ablation, *n* (%)	1 (2)	1 (6)	—	.28
Secondary prevention, *n* (%)	19 (29)	5 (28)	14 (30)	1.00
Ischemic cardiomyopathy, *n* (%)	40 (62)	11 (61)	29 (62)	1.00
Appropriate therapies, *n* (%)	19 (29)	3 (17)	16 (34)	.23
Single, *n* (%)	7 (11)	—	7 (14)	.18
During first year, *n* (%)	13 (20)	2 (11)	11 (23)	.33
Electrical storm, *n* (%)	4 (6)	1 (6)	3 (6)	1.00
Inappropriate therapies, *n* (%)	5 (8)	1 (6)	4 (9)	1.00
Mortality, *n* (%)	2 (3)	1 (6)	1 (2)	.49
LV‐EF, (%)	27 ± 8	29 ± 7	26 ± 8	.25
LA diameter, mm	50 ± 7	53 ± 7	49 ± 6	.026
LVEDD, mm	63 ± 9	63 ± 9	64 ± 9	.66
Biventricular pacing (BP), %	94 ± 7	93 ± 7	95 ± 6	.23
Responder, *n* (%)	41 (63)	13 (72)	28 (60)	.40
Heart rate at baseline, bpm	68 ± 18	66 ± 18	72 ± 18	.28
QRS duration, ms	147 ± 23	140 ± 23	148 ± 27	.35
Creatinin, mmol/l	112 ± 64	108 ± 23	111 ± 39	.65

Abbreviations: AF, atrial fibrillation; AV, atrio‐ventricular; CRT‐D, cardiac resynchronization therapy‐defibrillator; LA, left atrium; LV‐EF, left ventricular ejection fraction; LVEDD, left ventricular end‐diastolic diameter.

### 3.2 Predictors of permanent AF

**FIGURE 1:**
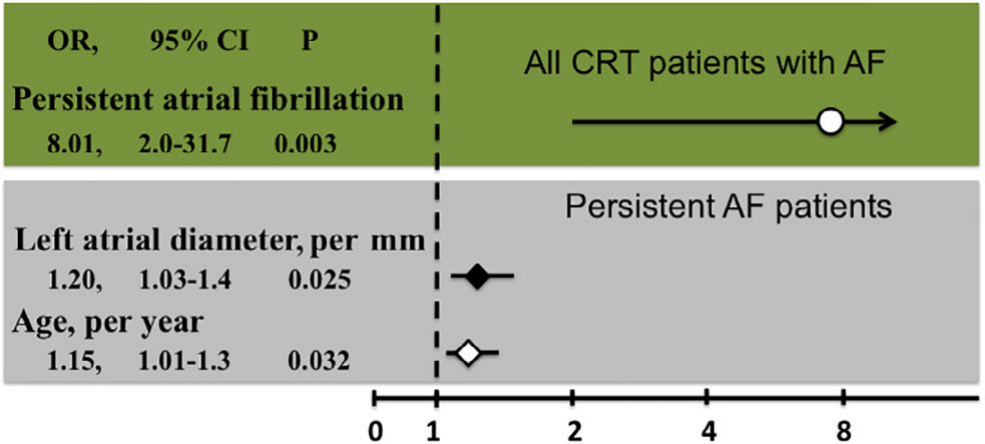
Logistic regression for the predictors of permanent atrial fibrillation (AF) in cardiac resynchronization therapy (CRT) patients. Graphical representation of the odds ratios (OR), the 95% confidence intervals (95% CI) and the *p* values

Patients who developed permanent AF had similar characteristics with the rest of the cohort (Table 1), but a higher incidence of persistent AF (83% vs. 38%, *p* = .002) and a larger LA diameter (53 ± 7 vs. 49 ± 6 mm, *p* = .026). The BP was similar between groups (Table 1), but less patients with permanent AF achieved BP of >92% (61% vs. 87%, *p* = .03) compared to those with non‐permanent AF. Logistic regression in the whole study population showed that persistent AF at the time of implantation (OR: 8.01, 95% CI: 2.0–31.7, *p* = .003) was the only significant predictor associated with progression to permanent AF. A subanalysis in persistent AF patients (Supplementary Table 1) revealed that a bigger LA diameter (OR: 1.2 per mm, 95% CI: 1.03–1.4, *p* = 0.025) and a higher age (OR: 1.15 per life‐year, 95% CI: 1.01–1.3, *p* = .032) were independent predictors of future permanent AF (Figure 1). At discrimination analysis, an LAD of 52 mm and an age of 71 years combined the highest specificity and sensitivity (Supplementary Figure 1). The combination of these criteria (persistent AF, LAD≥52 mm, age ≥ 71) differentiated patients that progressed to permanent AF with a specificity of 96%, a sensitivity of 45%, PPV of 80% and NPV of 82%. Patients with all criteria (*n* = 10) had a higher risk for continuous MS (OR: 18, 95% CI: 3.307–97.96, *p* = .001).

### 3.3 Predictors of stable SR

**FIGURE 2:**
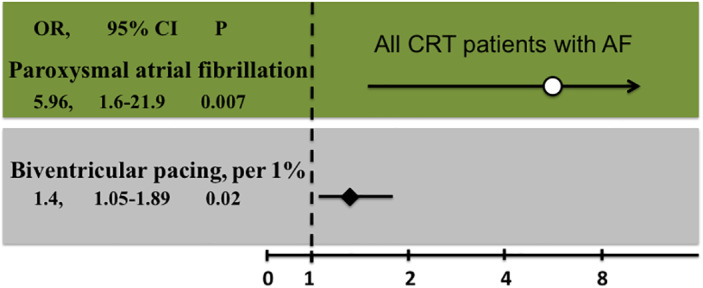
Logistic regression for the factors independently associated with a stable sinus rhythm (SR) during follow‐up in atrial fibrillation (AF) patients undergoing cardiac resynchronization therapy (CRT) implantation. Graphical representation of the odds ratios (OR), the 95% confidence intervals (95% CI) and the *p* values

Patients with stable SR (MS < 1%) during follow‐up had a higher incidence of paroxysmal AF at implantation (75% vs. 38%, *p* = .007) and a higher average BP (98 ± 2 vs. 92 ± 8%, *p* < .001) than the rest of the cohort (Table 2). Logistic regression showed that history of paroxysmal AF at the time of implantation (OR: 5.96, 95%CI: 1.6–21.9, *p* = .007) and an increased BP (OR: 1.4 per 1%, 95%CI: 1.05–1.89, *p* = .02) were associated with MS of less than 1% during follow‐up (Figure 2). From patients with persistent AF at implantation, those with stable SR during follow‐up (MS < 1%, *n* = 5) had higher BP compared to those with AF recurrence (98 ± 2 vs. 92 ± 8%, *p* < .001). Inclusion of the three patients without an atrial lead in our analysis did no significantly change the results of the study.

**TABLE 2:** Characteristics of CRT‐D patients with (mode switch, MS < 1%) and those with MS > 1% at follow‐up

	Total	Stable SR at follow‐up		*p*
Number of patients, *n*	65	Yes (20)	No (45)	
Age, (years)	69 ± 8	70 ± 9	68 ± 8	.42
Males, *n* (%)	51 (79)	16 (80)	35 (78)	.56
Body mass index, kg/m^2^	29 ± 8	28 ± 9	29 ± 8	.79
Diabetes mellitus, *n* (%)	22 (34)	10 (50)	12 (27)	.09
Hypertension, *n* (%)	54 (83)	15 (75)	39 (87)	.29
Persistent AF, *n* (%)	33 (50)	5 (25)	28 (62)	.007
NYHA class II, *n* (%)	23 (35)	5 (25)	18 (40)	.16
NYHA class III, *n* (%)	35 (54)	14 (70)	21 (47)	.17
NYHA class IV, *n* (%)	7 (11)	1 (5)	6 (13)	.89
Cardioversion, *n* (%)	34 (52)	8 (40)	26 (60)	.28
Amiodaron/Sotalol, *n* (%)	29 (45)	6 (30)	23 (51)	.12
LA ablation for AF, *n* (%)	6 (9)	1 (5)	5 (11)	.66
AV node ablation, *n* (%)	1 (2)	—	1 (2)	1.00
Secondary prevention, *n* (%)	19 (29)	6 (30)	13 (28)	1.00
Ischemic cardiomyopathy, *n* (%)	40 (62)	9 (45)	31 (69)	.10
Appropriate therapies, *n* (%)	19 (29)	4 (20)	15 (33)	.38
Single, *n* (%)	7 (11)	2 (10)	5 (11)	1.00
During first year, *n* (%)	13 (20)	4 (20)	9 (20)	1.00
Electrical storm, *n* (%)	4 (6)	—	4 (9)	.30
Inappropriate therapies, *n* (%)	5 (8)	1 (5)	4 (9)	1.00
Mortality, *n* (%)	2 (3)	—	2 (4)	1.00
LV‐EF, (%)	27 ± 8	26 ± 7	27 ± 8	.64
LA diameter, mm	50 ± 7	48 ± 7	51 ± 7	.14
LV‐EDD, mm	63 ± 9	63 ± 9	65 ± 9	.96
Biventricular pacing, %	94 ± 7	98 ± 2	92 ± 8	<.001
Responder, *n* (%)	41 (63)	13 (65)	28 (62)	.40
Heart rate at baseline, bpm	70 ± 18	71 ± 19	70 ± 19	.97
QRS duration, ms	147 ± 23	146 ± 27	146 ± 27	.98
Creatinin, mmol/l	112 ± 64	107 ± 38	112 ± 34	.57

Abbreviations: AF, atrial fibrillation; CRT‐D, cardiac resynchronization therapy‐defibrillator; LV‐EF, left ventricular ejection fraction; LVEDD, left ventricular end‐diastolic diameter.

## 4 DISCUSSION

### 4.1 Main findings

Our study shows that after CRT therapy in patients with history of AF, approximately one third will progress to permanent AF and continuous atrial mode‐switch (MS≥99%) and one third will achieve stable SR (MS < 1%). To the best of our knowledge, this is the first study to identify factors that could predict future rhythm outcomes and could assist the decision for the implantation of an atrial lead. We found that history of persistent AF, dilated left atrium (LAD≥52 mm) and advanced age (≥71) could predict a high risk for permanent AF (OR 18) with high specificity (96%) and PPV (82%). Paroxysmal AF at baseline and a higher BP during follow‐up are associated with improved rhythm outcomes and stable SR. These findings may help to design future prospective studies and improve the management of CRT patients according to baseline data.

### 4.2 CRT and chronic AF

Patients with AF and HF have increased morbidity and mortality and could benefit from a CRT with improvement of symptoms; systolic LV function and even survival.^4^

However, AF poses some challenges on CRT therapy because intrinsic AV conduction during AF episodes, upon exertion or even at normal rates may limit an adequate BP. Although the benefits of CRT in AF patients are comparable to those of SR patients, rhythm stability is not common and rate‐control drugs with adverse effects are needed.^5^, ^6^, ^7^ Kies et al. reported on patients with chronic AF, the majority of who (93%) remained in AF after 6 months of CRT, even those (13/18, 72%) who underwent successful cardioversion after implantation.^9^ In contrast, Lellouche et al. found that in patients without increased LA diameter and predominantly in SR, CRT could reduce AF burden.^8^ Gasparini and Kuperstein et al. have also emphasized the importance of LA size for subsequent clinical outcomes.^11^ Accordingly, the majority (73%) of the centers implant an atrial lead only if justified by the LAD and/or recent AF onset, some (14%) implant it in the hope of SR restoration, while others (4%) never implant an atrial lead.^16^ Criteria for patient selection though have never been adequately investigated.

In our study, one third of the patients progressed to permanent AF, from the very first follow‐up (at 4 weeks), limiting the usage of an atrial lead. AF type, age and LA size were the most important surrogates of advanced disease that were related with lower chances of rhythm stability. The combination of the three criteria proposed in this study (persistent AF, LAD≥52 mm and age ≥ 71 years) translated into a very high probability of 95% (OR 18) for constant MS. Thus in these patients, the use of an atrial lead is imposing additional complication risks without much expected benefit and could be avoided. This study therefore suggests that omitting the atrial lead in patients with these criteria is warranted without the need for a later procedure in most cases.

### 4.3 The role of biventricular pacing

Many studies suggest that CRT benefits are greatest in patients with very high levels of biventricular pacing. In the ALTITTUDE study, BP < 95% was associated with higher mortality.^17^ Similarly, the CRT RENEWAL & REFLEX trials showed that BP < 92% was associated with higher incidence of heart failure events.^18^ In AF patients with CRT though, the impact of BP on AF is not well studied yet. The present study emphasizes the rhythm‐stabilizing effects of CRT through the independent association and the positive bidirectional effect between a high BP and SR. Certainly the ongoing rhythm has a direct effect on BP but as seen here the BP during follow‐up is also associated with less AF. Thus, both BP and SR influence one another and do not follow a relationship of one‐way causality. Since BP is a variable obtained during follow‐up, it should not be seen as a predictor but rather as a factor associated with an antiarrhythmic effect and SR stability. As such, BP may not be suitable to guide decision early during follow‐up, but may indeed guide a more aggressive rhythm therapy later on.

In a meta‐analysis examining the effect of CRT on AF, the rate of conversion to SR was 10.7% (7–17%).^10^ In our study, a sufficient BP was achieved (94 ± 7%) and the majority of patients had a rhythm control attempt, allowing for 31% of the patients to maintain stable SR, despite the history of paroxysmal or persistent AF episodes in the months prior to CRT.

There is a dearth of data on factors distinguishing patients who derive an atrial antiarrhythmic effect from CRT. The only model currently available suggests that the conversion of permanent AF to SR is associated with LV‐EDD < 65 mm, LAD<50 mm, and AV node ablation.^19^ Consequently, AV node ablation has been suggested for patients with high AF burden and BP < 85%.^6^ Factors associated with CRT response in the MADIT‐CRT trial were not independently associated with an atrial antiarrhythmic effect.^20^ The present study supplements these data and underlies the atrial antiarrhythmic effect of effective BP (CRT). This is supplemented by a variety of rhythm strategies and can be achieved despite the tendency for less AV node ablation.^21^, ^22^ Recently the CASTLE‐AF trial by Marrouche et al. revealed that catheter ablation for AF in patients with heart failure was associated with a significantly lower rate of a composite end‐point of death or hospitalization for worsening HF. However, in this trial only 28% of the patients had CRT and subgroup analysis revealed a significant interaction for those with EF > 25% but not for the presence of CRT.^23^ In that sense, a more aggressive rhythm control strategy in our cohort may have drawn a different picture.

Additionally, recent developments in CRT therapy like His‐bundle or left‐bundle‐branch‐pacing demonstrated superior resynchronization and a trend toward higher echocardiographic response than biventricular CRT.^24^, ^25^ Such newer CRT techniques may have an improved impact on sinus rhythm stability. Thus, data from further CRT trials are warranted before the appropriate rhythm strategy is clear and the optimal BP cut‐off is defined.

### 4.4 Clinical implications

The present findings improve our understanding about the factors that impact rhythm stability in CRT‐AF patients. For the first time, we showed that persistent AF type, LA enlargement and age could predict future permanent AF. Our study suggests that patients with these criteria (persistent AF, LAD≥52 mm, ≥71 years) have a substantially increased risk (18‐fold) for permanent AF. On the other hand, patients with paroxysmal AF should aim for the highest BP possible in order to maximize the atrial antiarrhythmic effect of CRT therapy. These findings are in line with previous knowledge about AF and CRT and provide some suggestive cut‐offs for the evaluation of these patients and the facilitation of tailored diagnostic and therapeutic strategies.

### 4.5 Limitations

The current study is a single‐center limited by the inherent limitations of retrospective studies. A systematic rhythm control strategy was attempted, but was limited by medical (thrombi) and personal reasons in a minority of patients. However, we included consecutive patients with regular and thorough interrogations to obtain a comprehensive dataset. All included patients had an atrial electrode that allowed for continuous registration of AF burden. Although BP could have a protective effect, AVJ, and LA ablation were underutilized in this cohort. The majority (2/3) of the patients; those who achieved SR or progressed to permanent AF, were not interested for a more aggressive AF management (ablation), that could have led to better results. Furthermore, ventricular sense response during AF could lead to fusion and BP overestimation. CRT response in this study was defined as clinical improvement and did not reach statistical significance. Inclusion of more clinical or echocardiographic parameters (e.g., LV end‐diastolic volume) during follow‐up may have drawn a different picture, but it would not be helpful at the time of implantation. Finally, heart failure treatment in this study was not captured and did not include recent improvements like sacubitril/valsartan that could influence the results of the study. Although the significance of these findings will need further evaluation in prospective trials, the proposed criteria have shown a good differential value and should be seen as hypothesis generating that could serve the design of future studies that may randomize patients to different implantation or rhythm therapy strategies.

## 5 CONCLUSION

Despite the beneficial effects of CRT, approximately one third of patients with history of AF at the time of implantation will progress to permanent AF. Our findings suggest that patients with persistent AF, dilated LA (LAD≥52 mm) and advanced age (≥71 years) have a higher risk (OR 18) for permanent AF. On the other hand, in 31% of patients with paroxysmal atrial fibrillation a stable SR with correspondingly high BP could be maintained. These findings could facilitate the management of CRT in AF patients and guide rhythm or rate control therapy in order to maximize its effect on rhythm.

## CONFLICT OF INTEREST

The authors declare no potential conflict of interest.

## Supporting information


**Supplementary Figure 1** 

## Data Availability

The data that support the findings of this study are available from the corresponding author, upon reasonable request.

## References

[1] Kobe J , Wasmer K , Andresen D , et al. Impact of atrial fibrillation on early complications and one year‐survival after cardioverter defibrillator implantation: results from the German DEVICE registry. Int J Cardiol. 2013;168(4):4184‐4190. 23948108 10.1016/j.ijcard.2013.07.110

[2] Darma A , Nedios S , Kosiuk J , et al. Differences in predictors of implantable cardioverter‐defibrillator therapies in patients with ischaemic and non‐ischaemic cardiomyopathies. Europace. 2016;18(3):405‐412. 26056190 10.1093/europace/euv138

[3] Botto GL , Dicandia CD , Mantica M , et al. Clinical characteristics, mortality, cardiac hospitalization, and ventricular arrhythmias in patients undergoing CRT‐D implantation: results of the ACTION‐HF study. J Cardiovasc Electrophysiol. 2013;24(2):173‐181. 23130781 10.1111/jce.12023

[4] Molhoek SG , Bax JJ , Bleeker GB , et al. Comparison of response to cardiac resynchronization therapy in patients with sinus rhythm versus chronic atrial fibrillation. Am J Cardiol. 2004;94(12):1506‐1509. 15589005 10.1016/j.amjcard.2004.08.028

[5] Gasparini M , Leclercq C , Lunati M , et al. Cardiac resynchronization therapy in patients with atrial fibrillation: the CERTIFY study (cardiac resynchronization therapy in atrial fibrillation patients multinational registry). JACC Heart Fail. 2013;1(6):500‐507. 24622002 10.1016/j.jchf.2013.06.003

[6] Gasparini M , Auricchio A , Metra M , et al. Long‐term survival in patients undergoing cardiac resynchronization therapy: the importance of performing atrio‐ventricular junction ablation in patients with permanent atrial fibrillation. Eur Heart J. 2008;29(13):1644‐1652. 18390869 10.1093/eurheartj/ehn133 PMC2442164

[7] Ferreira AM , Adragao P , Cavaco DM , et al. Benefit of cardiac resynchronization therapy in atrial fibrillation patients vs. patients in sinus rhythm: the role of atrioventricular junction ablation. Europace. 2008;10(7):809‐815. 18511438 10.1093/europace/eun135

[8] Lellouche N , De Diego C , Vaseghi M , et al. Cardiac resynchronization therapy response is associated with shorter duration of atrial fibrillation. Pacing Clin Electrophysiol. 2007;30(11):1363‐1368. 17976100 10.1111/j.1540-8159.2007.00872.x

[9] Kies P , Leclercq C , Bleeker GB , et al. Cardiac resynchronisation therapy in chronic atrial fibrillation: impact on left atrial size and reversal to sinus rhythm. Heart. 2006;92(4):490‐494. 16159986 10.1136/hrt.2005.064816 PMC1860874

[10] Hess PL , Jackson KP , Hasselblad V , Al‐Khatib SM . Is cardiac resynchronization therapy an antiarrhythmic therapy for atrial fibrillation? A systematic review and meta‐analysis. Curr Cardiol Rep. 2013;15(2):330. 23299710 10.1007/s11886-012-0330-6 PMC3685144

[11] Kuperstein R , Goldenberg I , Moss AJ , et al. Left atrial volume and the benefit of cardiac resynchronization therapy in the MADIT‐CRT trial. Circ Heart Fail. 2014;7(1):154‐160. 24347664 10.1161/CIRCHEARTFAILURE.113.000748

[12] Kloosterman M , Rienstra M , Mulder BA , Van Gelder IC , Maass AH . Atrial reverse remodelling is associated with outcome of cardiac resynchronization therapy. Europace. 2016;18(8):1211‐1219. 26718536 10.1093/europace/euv382

[13] Ponikowski P , Voors AA , Anker SD , et al. 2016 ESC guidelines for the diagnosis and treatment of acute and chronic heart failure: the task force for the diagnosis and treatment of acute and chronic heart failure of the European Society of Cardiology (ESC)developed with the special contribution of the heart failure association (HFA) of the ESC. Eur Heart J. 2016;37(27):2129‐2200. 27206819 10.1093/eurheartj/ehw128

[14] Stiles MK , Fauchier L , Morillo CA , Wilkoff BL . 2019 HRS/EHRA/APHRS/LAHRS focused update to 2015 expert consensus statement on optimal implantable cardioverter‐defibrillator programming and testing. J Interv Card Electrophysiol. 2020;59(1):135‐144. 31960345 10.1007/s10840-019-00662-4 PMC7508744

[15] Kosiuk J , Nedios S , Darma A , et al. Impact of single atrial fibrillation catheter ablation on implantable cardioverter defibrillator therapies in patients with ischaemic and non‐ischaemic cardiomyopathies. Europace. 2014;16(9):1322‐1326. 24532559 10.1093/europace/euu018

[16] Bongiorni MG , Proclemer A , Dobreanu D , Marinskis G , Pison L , Blomstrom‐Lundqvist C . Preferred tools and techniques for implantation of cardiac electronic devices in Europe: results of the European heart rhythm association survey. Europace. 2013;15(11):1664‐1668. 24170423 10.1093/europace/eut345

[17] Hayes DL , Boehmer JP , Day JD , et al. Cardiac resynchronization therapy and the relationship of percent biventricular pacing to symptoms and survival. Heart Rhythm. 2011;8(9):1469‐1475. 21699828 10.1016/j.hrthm.2011.04.015

[18] Koplan BA , Kaplan AJ , Weiner S , Jones PW , Seth M , Christman SA . Heart failure decompensation and all‐cause mortality in relation to percent biventricular pacing in patients with heart failure: is a goal of 100% biventricular pacing necessary? J Am Coll Cardiol. 2009;53(4):355‐360. 19161886 10.1016/j.jacc.2008.09.043

[19] Gasparini M , Steinberg JS , Arshad A , et al. Resumption of sinus rhythm in patients with heart failure and permanent atrial fibrillation undergoing cardiac resynchronization therapy: a longitudinal observational study. Eur Heart J. 2010;31(8):976‐983. 20071325 10.1093/eurheartj/ehp572

[20] Goldenberg I , Moss AJ , Hall WJ , et al. Predictors of response to cardiac resynchronization therapy in the multicenter automatic defibrillator implantation trial with cardiac resynchronization therapy (MADIT‐CRT). Circulation. 2011;124(14):1527‐1536. 21900084 10.1161/CIRCULATIONAHA.110.014324

[21] Ganesan AN , Brooks AG , Roberts‐Thomson KC , Lau DH , Kalman JM , Sanders P . Role of AV nodal ablation in cardiac resynchronization in patients with coexistent atrial fibrillation and heart failure a systematic review. J Am Coll Cardiol. 2012;59(8):719‐726. 22340263 10.1016/j.jacc.2011.10.891

[22] Ousdigian KT , Borek PP , Koehler JL , Heywood JT , Ziegler PD , Wilkoff BL . The epidemic of inadequate biventricular pacing in patients with persistent or permanent atrial fibrillation and its association with mortality. Circ Arrhythm Electrophysiol. 2014;7(3):370‐376. 24838004 10.1161/CIRCEP.113.001212

[23] Marrouche NF , Brachmann J , Andresen D , et al. Catheter ablation for atrial fibrillation with heart failure. N Engl J Med. 2018;378(5):417‐427. 29385358 10.1056/NEJMoa1707855

[24] Upadhyay GA , Vijayaraman P , Nayak HM , et al. On‐treatment comparison between corrective his bundle pacing and biventricular pacing for cardiac resynchronization: a secondary analysis of the his‐SYNC pilot trial. Heart Rhythm. 2019;16(12):1797‐1807. 31096064 10.1016/j.hrthm.2019.05.009

[25] Wu S , Su L , Vijayaraman P , et al. Left bundle branch pacing for cardiac resynchronization therapy: nonrandomized on‐treatment comparison with his bundle pacing and biventricular pacing. Can J Cardiol. 2020. 10.1016/j.cjca.2020.04.037 32387225

