# Supplementary figures and images for: Predictors of rhythm outcomes after cardiac resynchronization therapy in atrial fibrillation patients: When should we use an atrial lead?
Source: Clin Cardiol. 2020 Dec 9;44(2):210–7. doi: 10.1002/clc.23527 (PMC7852157; doi:10.1002/clc.23527)

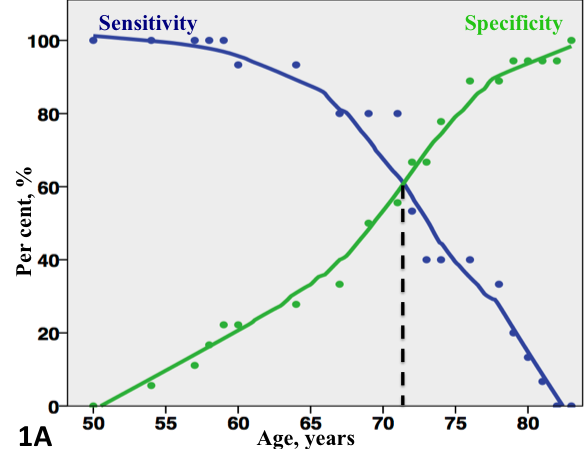

Supplement: Supplementary file 1 — Supplementary Figure 1 [file CLC-44-210-s001.zip › CLC_23527_Sup. Figure 1A.tiff]

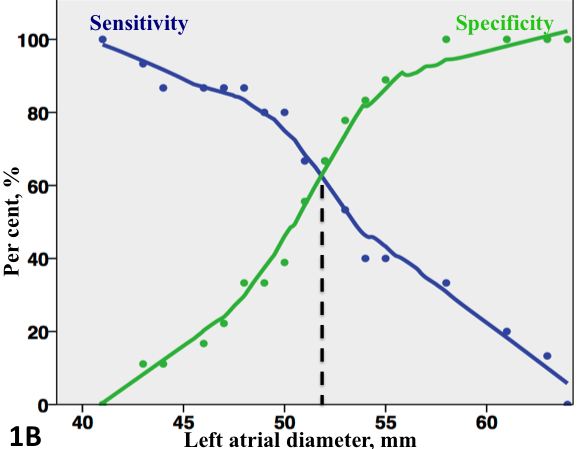

Supplement: Supplementary file 1 — Supplementary Figure 1 [file CLC-44-210-s001.zip › CLC_23527_Sup. Figure 1B.tiff]
